# PET/SPECT Molecular Probes for the Diagnosis and Staging of Nonalcoholic Fatty Liver Disease

**DOI:** 10.1177/1536012119871455

**Published:** 2019-09-03

**Authors:** Tuo Shao, Lee Josephson, Steven H. Liang

**Affiliations:** 1 Division of Nuclear Medicine and Molecular Imaging, Department of Radiology, Massachusetts General Hospital and Harvard Medical School, Boston, MA, USA

**Keywords:** biomarker, hematology

## Abstract

Nonalcoholic fatty liver disease (NAFLD) is a significant public health challenge afflicting approximately 1 billion individuals both in the Western world and in the East world. While liver biopsy is considered as gold standard in the diagnosis and staging of liver fibrosis, noninvasive imaging technologies, including ultrasonography, computed tomography, single-photon emission computed tomography (SPECT), magnetic resonance imaging, and positron emission tomography (PET) could offer more sensitive, comprehensive, and quantitative measurement for NAFLD. In this review, we focus on recent development and applications of PET/SPECT molecular probes that enable multispatial/temporal visualization and quantification of physiopathological progress at the molecular level in the NAFLD. We shall also discuss the limitations of current radioligands and future direction for PET/SPECT probe development.

## Introduction

Although the molecular pathways that lead to the pathogenesis and process of nonalcoholic fatty liver disease (NAFLD) and nonalcoholic steatohepatitis (NASH; a progressive form of NAFLD) remain poorly understood, it is accepted that inflammation, accumulation of extracellular matrix proteins, and proliferation of myofibroblasts are significant risk factors in hepatic injury.^1^ In the past decade, research has focused on the molecular mechanisms involved in the development from hepatic steatosis to more advanced hepatic inflammation and fibrosis. A variety of different signaling pathways and specific biomarker phenotypes provide solid molecular biology basis for the development of hepatic molecular imaging tools. At present, liver biopsy is the gold standard for diagnosing liver disease and assessing the stage of fibrosis, but several limitations and/or adverse effects are associated with this invasive procedure, including pain, severe complications, and sampling error due to heterogeneous lesion distribution.^2^ All these challenges present a unique opportunity for noninvasive methods for diagnosis and staging of NAFLD using translational molecular probes. Unlike ex vivo biopsy tests and histological testing, positron emission tomography (PET) / single-photon emission computed tomography (SPECT) imaging biomarkers enable direct characterization, quantification, and multispatial visualization of biological and cellular processes at different stages and the whole organism level. This approach would allow us to study a broad spectrum of physiological processes with the signal pathway, including proliferation, inflammation, and apoptosis, and evaluate various disease stages that represent the hallmarks of NAFLD.^3^ In addition, these different biomarkers can be further used in more precise functional evaluation complementary to the current metabolic imaging. In this work, we give a brief review of PET/SPECT imaging biomarkers and their applications in the monitoring and staging of NAFLD and future perspectives in the radioligand development.

### Prevalence and Progression of NAFLD

**Figure 1.**
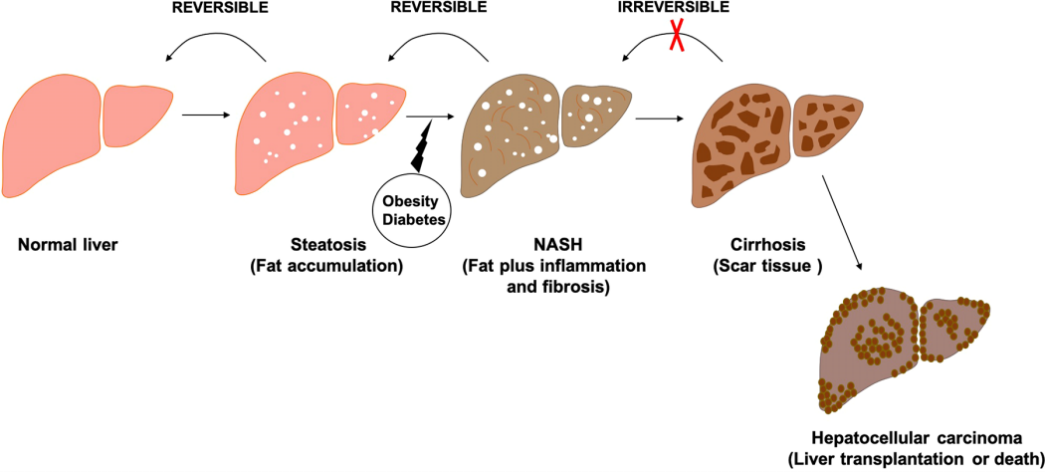
Progression of nonalcoholic fatty liver disease (NAFLD).

Nonalcoholic fatty liver disease is a prevalent type of chronic liver disease in both developed and underdeveloped countries. There is a high prevalence of NAFLD among those who have obesity, insulin resistance, cardiometabolic alterations, pattern, and metabolic syndrome. In developed countries, estimates of NAFLD prevalence vary between 20% and 30%, rising to 90% in morbidly obese populations. The more advanced form of NAFLD, NASH, carries a high risk of progressive fibrosis and cirrhosis and eventually developed to hepatocellular carcinoma (HCC).^4^ Type 2 diabetes is closely associated with NAFLD—70% of patients having steatosis with type 2 diabetes—and thus it is now recognized to represent the hepatic indication of metabolic syndrome.^5^ As shown in Figure 1, NAFLD encompasses a broad range of hepatic pathology from simple fat accumulation (steatosis) to hepatic inflammation or fibrosis (NASH) and finally cirrhosis and even HCC. Although there is no inflammation and other symptoms in the steatosis stage, approximately 20% of patients with steatosis will continue to develop NASH. Nonalcoholic steatohepatitis occurs when there is persistent scar tissue in the liver, when the scar tissue starts to replace normal tissue, leading to cirrhosis. At the cirrhosis stage, the majority of liver function is significantly impaired, causing a high risk of HCC.^5^

### Pathogenesis of NAFLD

Although the pathogenesis of NAFLD/NASH is still poorly understood, the most accepted concept about the pathogenesis of NAFLD involves multiple “hits.” These hits might promote isolated steatosis, innate immune activation, inflammation, cell death, or progressive liver damage.

#### Inflammation

An accepted concept of NAFLD pathogenesis involved a “2-hit” process, in which the abnormal metabolic environment is causing lipid accumulation comprised of the “first hit,” and this hit increases the susceptibility of the liver to secondary injuries (“second hit”) in inflammation.^6^ The severe consequences include mitochondrial dysfunction, overproduction, and the release of proinflammatory cytokines and chemokines, which notably include macrophage chemotactic protein 1, tumor necrosis factor α (TNF-α), interleukin (IL) 1β, and IL-1.^7^

#### Hepatocellular injury and cell death

Fibrogenesis occurs through phagocytosis when clearing apoptotic debris. During apoptosis, cells are divided into several small fragments, namely, apoptotic bodies.^8^ The “professional” hepatic phagocytes, such as Kupffer cells (stellate macrophages) and hepatic stellate cells (HSCs), perform an important role in the clearing of apoptotic bodies.^9^ The phagocytosis of apoptotic bodies is not merely a “clean-up” process to clear cellular corpses. Instead, phagocytosis initiates intracellular signaling transduction in the phagocyte, which leads to discrete immune responses including cytokines/chemokines generation and induces collagen type I, a biomarker of the cirrhotic scar. Especially, transforming growth factor β (TGF-β) is activated when cells engulf apoptotic bodies. The TGF-β is also a robust fibrogenic signal marker in the liver.^10^

#### Necrosis

Necrosis is a severe inflammatory mode of cell death compared to apoptosis. Necrosis occurs under several different conditions in the progression of hepatic diseases: adenosine triphosphate inhibition as a consequence of mitochondrial dysfunction; drug- or toxin-induced liver injury by xenobiotics; overgeneration of reactive oxygen species as it occurs during ischemia/reperfusion injury; and continual tissue injury as it occurs in chronic liver failure. The cell death model by noxious stimuli usually depends on the concentration, with low levels likely to lead to apoptosis and high levels inducing to necrosis. Therefore, the abovementioned stimuli might induce both modes of cell death at different time points, which depends on the severity of the injury.^11^

### Traditional Diagnostic and Staging Methods for NAFLD

#### Biopsy

At present, liver biopsy is the gold standard for diagnosing liver disease and assessing the stage of fibrosis. In the absence of efficacious interventions for the chronic liver disease, liver biopsy allows rapidly to evaluate the histological components and identify their relationship with disease. Indeed, biopsy provides essential information for the diagnosis of NASH based on the presence of steatohepatitis. The histopathological range of NASH involves 4 major factors: steatosis, inflammation, hepatocellular injury, and fibrosis. Another significant advantage of liver biopsy is to provide an accurate semiquantitative evaluation of the severity of damage.^12^ However, several limitations and drawbacks are present for this invasive procedure, such as pain, the reluctance of patients, and the risk of severe complications, and is subject to sampling error. Importantly, the person who performs it substantially determines the quality of the procedure. A successful procedure highly depends upon a well-trained hepatologist with sufficient experience.

#### Noninvasive assessment

Recently, several clinical markers have been utilized for predicting NASH and fibrosis. For example, the NAFLD fibrosis score is a scoring system to predict the advanced fibrosis based on several clinical parameters, such as age, body mass index, hyperglycemia, platelet count, albumin, and Aspartate aminotransferase (AST) - Alanine aminotransferase (ALT) ratio. When most of the biomarkers and scoring systems show similar accuracy for the detection of severity fibrosis, it is difficult to achieve high sensitivity for the diagnosis of early/mild fibrosis. False-positive results may also be caused by upregulation in bilirubin, decrease in haptoglobin, Gilbert syndrome, and cholestasis. In this context, noninvasive imaging techniques could be advocated as alternatives and diagnostic tests for NAFLD. Ultrasound-based transient elastography or FibroScan has shown prospective results for determination of the severity of liver fibrosis and degree of steatosis. However, it is also challenging in obese patients or those with ascites due to limited signal penetration as well as inadequate quantification of monitoring reversion in fibrosis after treatment.^2^ In this context, nuclear imaging techniques including SPECT and PET targeted with novel signaling pathways may show target-specific and biological characterization in viability and metabolic activity in the progression of NAFLD.^13^

## The Application of PET and SPECT Molecular Probes in the Diagnosis and Staging of NAFLD

### Preclinical Models for Imaging NAFLD

Animal models of NAFLD are essential in investigating the pathophysiological mechanisms of liver dysfunctions and diseases. To better our understanding of molecular targets that are associated with NAFLD, continuous efforts are contributed to the development of a variety of animal models to mimic the onset and progression of this process. There are currently several representative animal models of NAFLD, including NASH that develops due to high-fat dietary and essential nutrition factors defects and liver fibrosis induced by chemicals and based on surgery. The methionine and choline-deficient (MCD) diet is one of the frequently used and best described dietary models for NASH.^14^ The MCD diet usually contains a high sucrose content (eg, 40%) and moderate amounts of fat content (10%) but is short of methionine and choline. Attributed to the elevated intake of fatty acids, rodents with an MCD diet usually develop hepatic steatosis, followed by necrosis and inflammation, eventually to pericellular and pericentral fibrosis.^15^ Nuclear imaging of liver fibrosis has so far relied upon 2 models of chemically induced liver fibrosis and 1 surgical model, namely, carbon tetrachloride (CCl4) model, thioacetamide (TAA) model, and bile duct ligation (BDL) model. The CCl4 induces oxidative stress and necrotic response in the liver, generating toxic lipid and protein peroxidation products.^16^ Chronic CCl4 administration produces extensive liver damage with necrotic hepatocytes, degenerated and ballooned, as well as features of macro- and microsteatosis and mild mononuclear cell infiltration in the affected areas. The TAA also induces oxidative stress by the oxidation of its sulfur species to the corresponding sulfur oxides and dioxides, leading to hepatic cytochrome P450 enzyme-linked hepatoxicity.^17^ Bile duct ligation is the most common model used to induce obstructive cholestatic liver injury by surgical manipulation of bile acid circulation, which generates rapid-onset experimental hepatic fibrosis. Bile acids lead to dysfunction of farnesoid X receptor, liver X receptor, pregnane X receptors, and/or G-protein-coupled receptor TGR5, which are involved in a variety of metabolic and hepatic functions.^18^ Excess bile acids accumulation leads to, in order of increasing severity in liver dysfunction, acute oxidative stress, necroinflammation, fibrosis, cirrhosis, and end-stage liver failure.

### Translocator Protein 18 kDa as a Molecular PET Imaging Biomarker for Liver Fibrosis

**Figure 2.**
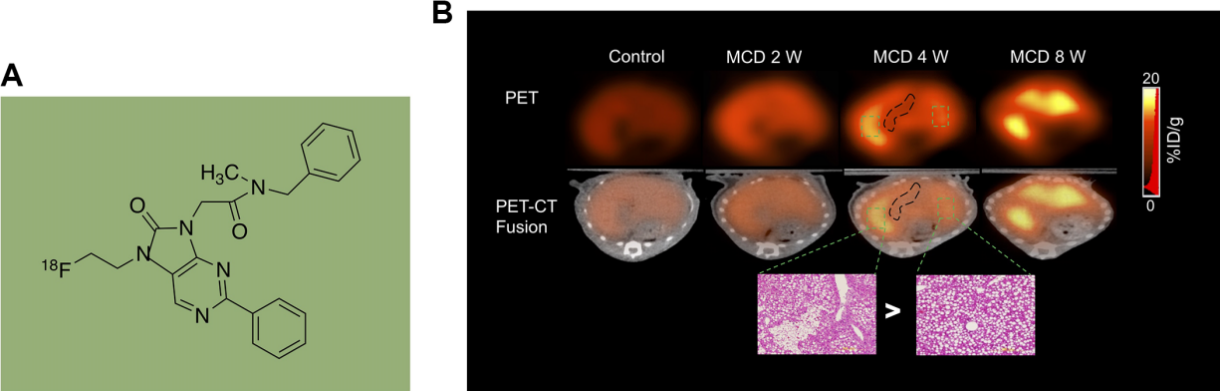
(A) Structure of [18F]FEDAC and (B) representative positron emission tomography (PET)/computed tomography (CT) images of the livers in methionine and choline-deficient (MCD) and control mice. (Courtesy of Dr Ming-Rong Zhang).

**Figure 3.**
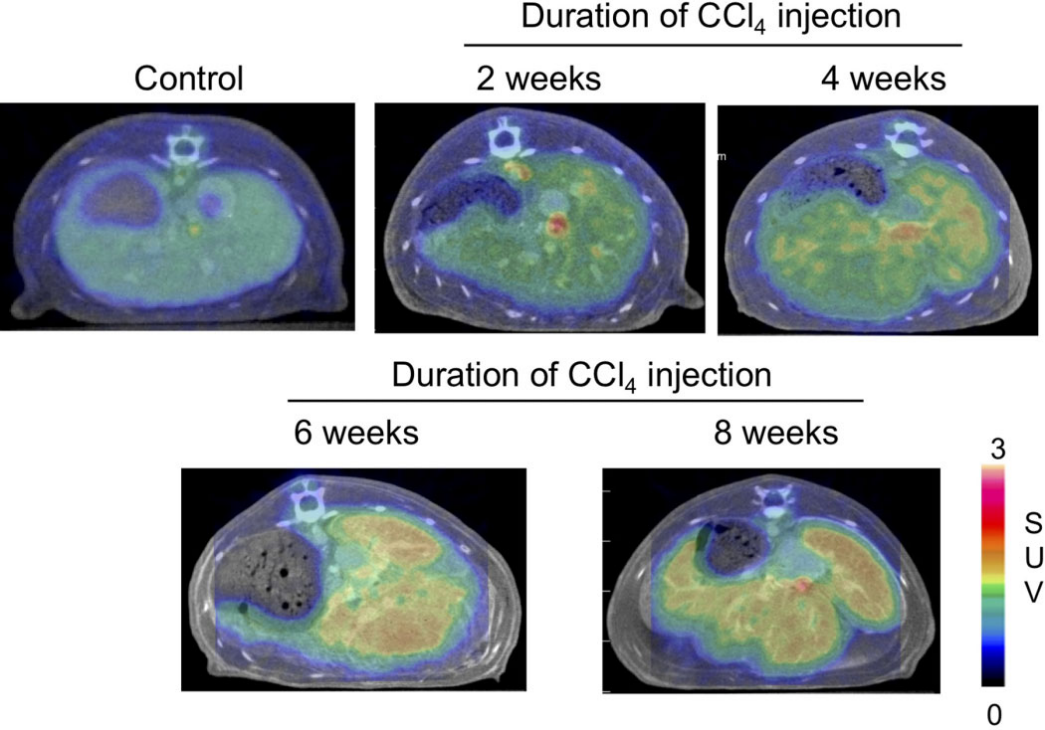
Representative transverse positron emission tomography (PET)/computed tomography (CT) fusion images of control and carbon tetrachloride (CCl4)-treated rat livers. (Courtesy of Dr Ming-Rong Zhang).

Translocator protein 18 kDa (TSPO), a nucleus-encoded mitochondrial target transmembrane protein, has been indicated to play an essential role in the regulation of mitochondrial function and is increased in the inflammatory cells.^19^ Overexpression of TSPO is a hallmark of inflammation. Elevated expression of this protein is reported not only during NAFLD but also in reactive retinal microglia and in a rat model of focal cerebral ischemia, which was imaged using (18)F-DPA-714 PET tracer.^20^ Inflammation occurs during hepatic fibrogenesis. During the progression of liver fibrosis, activated HSCs (aHSCs) generated high TSPO expression, which may accelerate hepatic fibrogenesis. Moreover, TSPO expression has been shown in transformed HSC both in vitro and in vivo, and TSPO is also an inflammation biomarker in PET imaging.^21,22^ Thus, TSPO is a useful biomarker to monitor the progression of liver fibrosis using specific PET radiotracers. From histopathology and autoradiography studies, Xie et al recently demonstrated that [^18^F]FEDAC (Figure 2A) uptake was significant from injured hepatocytes and the necroinflammatory loci of CD11b+ macrophages in a mouse model of MCD diet-induced NAFLD^23^ (Figure 2B). These results suggested that inflammation may also be involved in NAFLD process, and [^18^F]FEDAC may be a potential imaging tracer for NAFLD. In another mouse model of hepatic fibrosis, Hatori et al demonstrated that TSPO-specific radioligand [^18^F]FEDAC provided noninvasive visualization of the progression from fibrosis to cirrhosis.^24^ The experimental model of this study involved the induction of hepatic fibrosis by CCl_4_ exposure. This study showed significant uptake was mainly from HSCs and TSPO-expressing macrophage in 8-week CCl_4_ group (44.2 ± 0.7, standardized uptake value [SUV] × minute) compared to the control group (29.0 ± 0.3, SUV × minute; Figure 3). Their results also confirmed the distribution of bound radioactive signals was associated with TSPO binding in the fibrotic liver. The messenger RNA expression level of liver TSPO and associated TGF-β1, PDGF-β, and TNF-α were upregulated by 6 weeks of CCl_4_ treatment. Level of TSPO was correlated positively with the expression of proinflammatory cytokine factors. In summary, these studies demonstrated that TSPO is highly expressed and accurately reflects the histological figure of NAFLD/NASH in murine models. [^18^F]FEDAC showed a sensitive and specific visualization and quantification during liver steatosis and fibrosis progression; it may be useful to help build a reliable, noninvasive method for imaging NAFLD.

### Integrin α_v_β_3_ Targeted Imaging Studies

**Figure 4.**
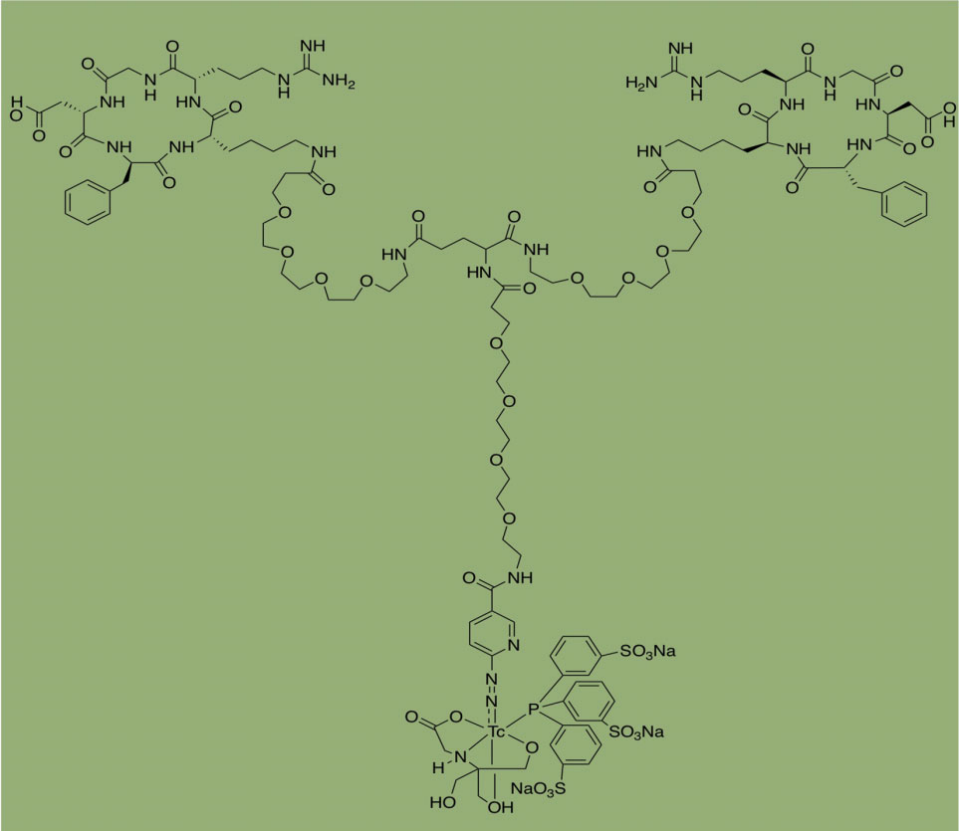
Structure of [^99m^Tc]3PRGD_2_.

Integrins comprise many cell surface receptors by α and β subunits, and each αβ combination has its binding specificity and signal transduction pathway.^25^ The integrin α_v_β_3_ is the major adhesion receptor that reacts to the ECM and thus plays an important role in control cell migration, proliferation, differentiation, and apoptosis, and dysregulated integrin αvβ3 receptor is another exciting target for NAFLD diagnosis/staging and cancer theranostics.^26^ A common characteristic of integrins family, including α_v_β_3_, binds to ECM proteins by way of targeting arginine–glycine–aspartate (RGD) tripeptide sequence, a ligand that was previously used in cancer imaging and drug delivery.^27^ Therefore, several cyclic RGD peptides (cRGD) are radiolabeled and developed as PET/SPECT tracers for binding integrin α_v_β_3_-positive targets.^28-30^ Liver fibrosis studies have shown that integrin α_v_β_3_ exhibits high expression of aHSCs and promotes HSC survival and proliferation.^31^ Li et al systemically detected the applicability of ^99m^Tc-labeled cRGDfK for SPECT imaging of HSC activity in fibrotic livers of TAA-treated rodent models.^32^ The normal, moderate fibrotic (TAA treatment for 3 weeks) or severe fibrotic livers (9 weeks TAA treatment) could be distinguished by the mean radioactivity ratio of the liver to heart (MRAR) under SPECT imaging using [^99m^Tc]cRGDfK. Coadministration of cold cRGDfK can successfully block [^99m^Tc]cRGDfK uptake in the fibrotic liver; this confirmed the specificity of cRGDfk for liver uptake. Expression levels of integrin α_v_ and β_3_ subunits were enhanced with the progression of liver fibrosis and decreased with its regression. These results demonstrated that [^99m^Tc]cRGDfK was associated with α_v_β_3_ binding during the fibrotic liver disease. The binding affinity of integrin α_v_β_3_ can be further improved through using dimeric or multimeric cRGD peptides. Zhang et al further used [^99m^Tc]3PRGD_2_ (Figure 4) in the TAA-induced liver fibrosis model of rats.^33^ The radiotracer was bound specifically with the integrin α_v_β_3_ mainly expressed on the aHSCs. The MRAR was increased in the fibrotic livers compared to that of controls (TAA, 1.98 vs control, 1.50) at 30 minutes postinjection. The liver t_1/2_ was longer than in the controls (TAA, 27.07 ± 10.69 minutes vs control, 12.67 ± 4.10 minutes). Another work by Yu et al demonstrated [^99m^Tc]3PRGD_2_ was not only used to monitor the progression of liver fibrosis but also to measure the decrease in [^99m^Tc]3PRGD_2_ uptake in the fibrotic liver after antifibrotic therapy with drug interferon α2b.^34^

### Asialoglycoprotein Receptors Imaging for Hepatic Fibrosis

**Figure 5.**
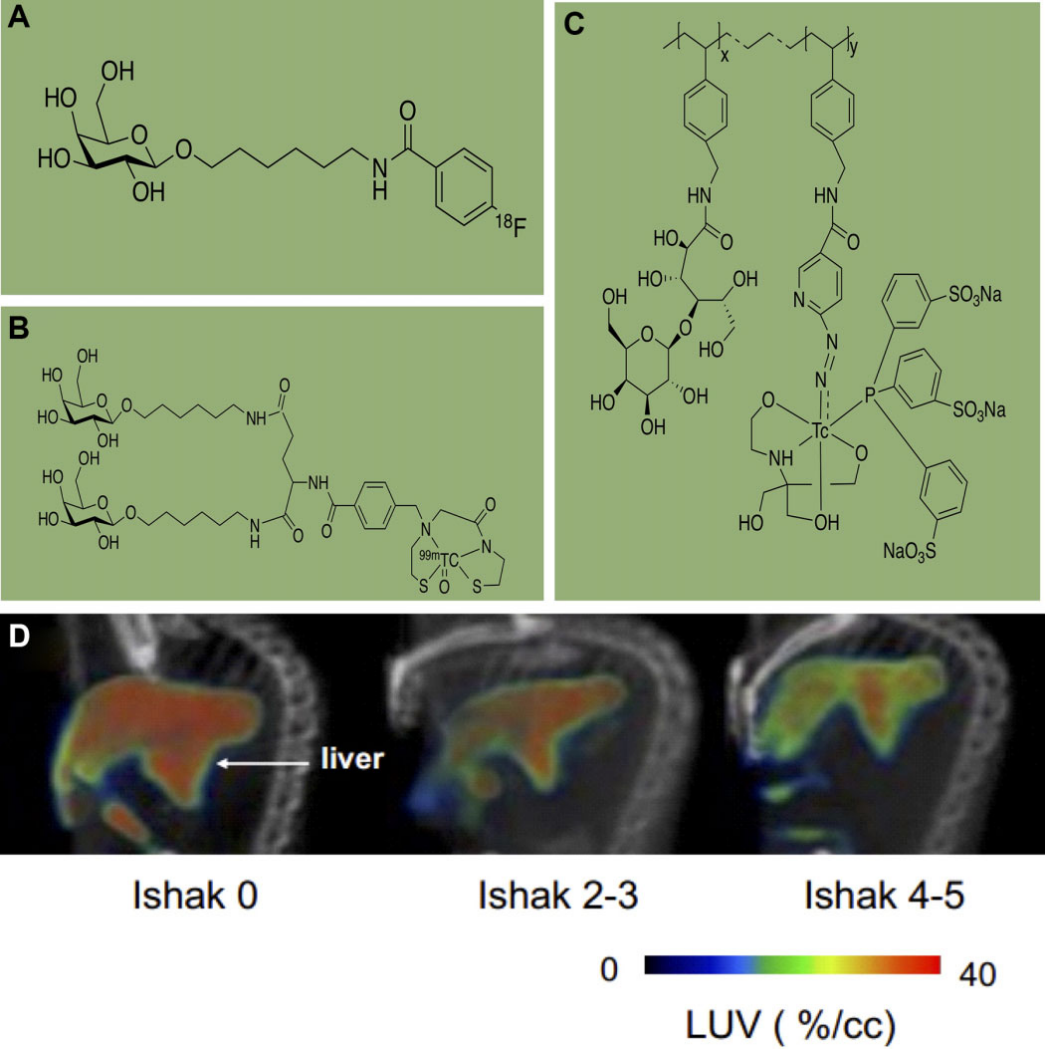
Structure of (A) [18F]FBHGal, (B) [99mTc]MAMADGal, (C) [99mTc]p(VLA-co VNI) and single-photon emission computed tomography (SPECT)/computed tomography (CT) images of control and carbon tetrachloride (CCl4)-induced fibrotic mice show the liver. (Courtesy of Dr Xianzhong Zhang).

Asialoglycoprotein receptors (ASGPRs) are well known to colocalize with the mammalian liver, of which 90% exists on sinusoidal faces of hepatocytes and only 10% on lateral faces.^35,36^ The expression of ASGPR on the hepatocytes of patients with liver disease is reduced and recognized as a diagnostic biomarker for the evaluation of liver function. The previous study demonstrated ASGPR activity in the patients with cirrhotic liver was decreased to about 28% compared to the healthy controls.^37,38^ [^18^F]FBHGal is a monovalent galactose derivative that was radiolabeled with fluorine-18 as ASGPRs-specific PET probe. Recent studies also reported several galactosides and *N*-acetyl galactosamine derivatives, including mono- and multivalent ligands, as potential substrates for ASGPRs in vitro. Kao et al performed biodistribution studies of [^18^F]FBHGal in both normal and dimethylnitrosamine-induced hepatic fibrosis mouse models.^39^ In both studies, the receptor indexes (liver/liver plus heart ratio at 30 minutes postinjection) of hepatic fibrosis mice were significantly lower (*P* < .01) compared to those of normal mice, and the accumulation of [^18^F]FBHGal in fibrosis liver (∼15%ID/g) was decreased compared to normal liver (∼21%ID/g) at 30 minutes postinjection. The protein expression level of hepatic ASGPRs in the liver fibrosis mouse was significantly decreased compared to that of normal mice. The results indicate that [^18^F]FBHGal (Figure 5A) is a feasible agent for PET imaging of liver fibrosis. At the same time, Chang et al also have demonstrated that [^99m^Tc]MAMA-DGal is an ASGPRs-specific SPECT imaging probe for monitoring hepatic fibrosis.^40^ The authors synthesized ^99m^Tc-labeled divalent galactosides, [^99m^Tc]MAMA-DGal (Figure 5B), and performed SPECT imaging and biological characterization in normal and liver fibrosis mouse model. [^99m^Tc]MAMA-DGal provides significant specific binding to ASGPRs in normal liver than fibrosis and then rapidly excreted through both renal clearance and hepatobiliary system. Therefore, [^99m^Tc]MAMA-DGal could be used to reveal liver images and provide quantitative results for ASGPRs-related liver dysfunction. Recently, a new synthetic copolymer [^99m^Tc]*p*(VLA-co-VNI) was (Figure 5C) reported by Zhang et al and imaging studies of [^99m^Tc]p(VLA-co-VNI) can identify different stages of liver fibrosis, which is CCl_4_-induced liver fibrosis in mouse models.^41^ The authors first demonstrated ASGPR expression correlated with liver fibrosis progression. The liver uptake value (LUV) decreased along with the disease progression (control: 25.5 ± 1.58, 4 weeks of CCl_4_: 19.0 ± 2.12, 8-12 weeks of CCl_4_: 14.3 ± 2.41; Figure 5D). After the antifibrotic Tan IIA treatment, LUV increased nearly 200% than the control group, which was then confirmed by the Sirius Red staining and hydroxyproline analysis.

### Galactosyl Human Serum Albumin for Staging Fibrosis in NASH

**Figure 6.**
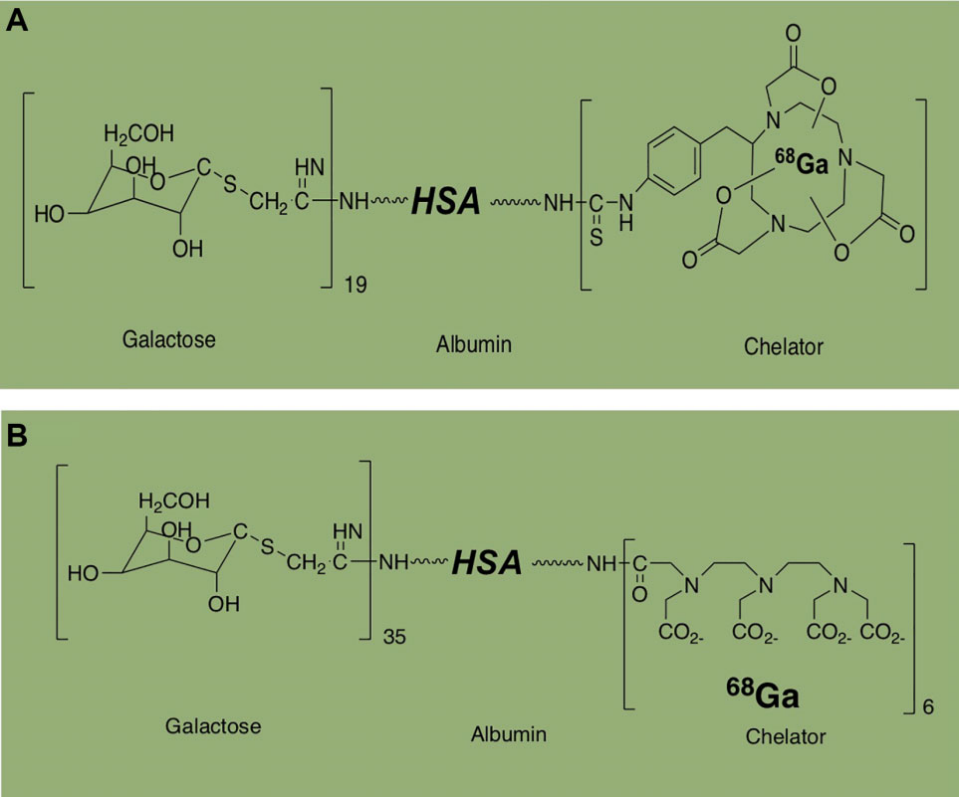
Structure of [^68^ Ga]NOTA-GSA and [^68^ Ga]DTPA-GSA.

Galactosyl human serum albumin (GSA) is a synthetic analog ligand of ASGPRs. ^99m^Tc-labeled galactosyl human serum albumin ([^99m^Tc]GSA) has shown that it binds specifically to the ASGPR and allows estimation of regional hepatic function and the progression of chronic viral hepatitis in preclinical and clinical studies.^42,43^ Haubner et al developed a ^68^Ga-labeled analog^44^ for PET imaging studies (Figure 6A). [^68^Ga]NOTA-GSA showed a significant increase in the metabolic stability in the liver and had a lower background activity in other organs. Schnabl et al performed ^68^Ga-labeled DTPA-conjugated neogalactosyl human serum albumin ([^68^Ga]DTPA-GSA; Figure 6B), using T_90_ values to characterize [^68^Ga]GSA uptake in monitoring hepatic fibrosis and progression of NASH in rats.^45^ In their PET imaging studies, animals with dominant pattern F0 (a rat with a healthy liver) to F1 (a rat with early or mild fibrosis) demonstrated significantly faster accumulation of [^68^Ga]GSA (*T*_90_: 2.40 ± 0.52 minutes) than those with moderate to advanced dominant pattern fibrosis F2 (moderate fibrosis) and F4 (cirrhotic liver; *T*_90_: 3.48 ± 1.01 minutes). These results demonstrated [^68^Ga]GSA accurately distinguishes early from mild experimental fibrosis independent of steatosis grade.

### Targeting Desmin and Vimentin for aHSCs

**Figure 7.**
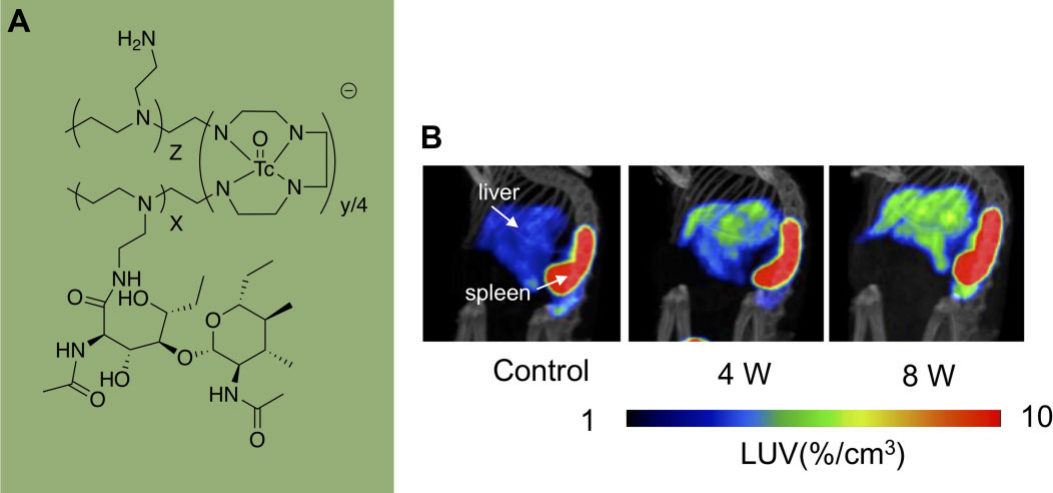
(A) Structure of [99mTc]GlcNAc-PEI and (B) single-photon emission computed tomography (SPECT)/computed tomography (CT) imaging of carbon tetrachloride (CCl4)-induced fibrotic mice with [99mTc]GlcNAc-PEI. (Courtesy of Dr Xianzhong Zhang).

Desmin and vimentin are members of type III intermediate filament protein family and present in both muscle and nonmuscle cells. The expression of both vimentin and desmin is substantially increased during HSCs activation, and its protein level is higher than quiescent HSCs.^46,47^ GlcNAc has a high affinity for desmin and vimentin; therefore, radiolabeled polyethylenimine-1800 (PEI-1800) modified GlcNAc can be used for targeting the HSCs. Zhang et al utilized [^99m^Tc]GlcNAc-PEI (Figure 7A) to assess liver fibrosis in a CCl_4_-induced liver fibrosis mouse model.^48^ The [^99m^Tc]GlcNAc-PEI imaging study showed the LUV in 8-week CCl_4_ treatment group was higher than that of 4-week group (4.7%/cc vs 3.3%/cc) and significantly increased compared to the control group (4.68%/cc vs. 2.34%/cc; Figure 7B). In vivo imaging results were confirmed by ex vivo biodistribution studies. In addition, [^99m^Tc]GlcNAc-PEI was used to detect the therapeutic efficacy of liver fibrosis progression. After clodronate liposomes treatment, [^99m^Tc]GlcNAc-PEI uptake was reduced in fibrotic mice (control vs clodronate: 4.62%/cc vs 2.13%/cc). These results demonstrated [^99m^Tc]GlcNAc-PEI is a potential tracer to detect fibrosis progression and monitor the treatment of anti-fibrotic drugs.

## Conclusion and Future Perspectives

Over the past decade, there has been considerable progress focusing on the cellular and molecular mechanisms involved in the progression of NASH to fibrosis. The research progress of different signaling pathways and specific biomarker expression promote the development of hepatic molecular imaging (Table 1). Although several PET/SPECT-specific molecular probes, particularly, radioactive molecules targeting TSPO, α_v_β_3_, ASGPR, GSA, and desmin/vimentin, have been used in the PET studies of liver fibrosis in animal experiments, there is no PET/SPECT tracer available for human use. Detection of the activation, transformation, and proliferation of myofibroblasts in the early stage of liver fibrosis is still challenging, which represents an unmet and urgent clinical need for imaging studies. Several factors should be considered when applying a PET/SPECT tracer for clinical translation. First, the liver is recognized as one of the most complex organs and contributes most metabolic activities in the human body. The liver consists of different cell types, including HSCs, Kupffer cells, sinusoidal endothelial cells, and hepatocytes, and the activation of HSC is the primary effector cell during liver fibrosis progress. Although TSPO, integrin αvβ3, desmin, and vimentin have high expression in aHSCs, these targets probes also showed nonspecific uptake by other cells, which contributed low signal to background ratio particularly in the early phase of PET/SPECT scans. Therefore, radiotracer labeled with relatively long half-life isotopes, such as ^18^F and ^64^Cu, and/or excretion mainly via a nonhepatic pathway to reduce background uptake and dosimetry, for example, renal clearance, may facilitate the development of new imaging probes for liver dysfunctions. It is also essential to design clinical trials and human translation research combined with emerging targeted therapy drugs for liver diseases and use PET/SPECT as companion readouts for target engagement and assessment for treatment efficacy. It will also be advantageous to identify individuals at high risk of disease progression and stratify patients who would likely benefit from specific targeted therapy (patient selection). Recently, there are several promising therapeutic/diagnostic targets that may provide an alternative approach for PET/SPECT probe development. To name a few, Caravan et al developed a type I collagen–targeted PET probe for detecting and staging pulmonary fibrosis.^49^ The same probe may provide a translational tool for patients with liver fibrosis and other fibrotic diseases. The endocannabinoid system plays a crucial role in acute and chronic liver injury. Numerous studies in animal models of NAFLD^50-56^ have implied that CB1 and CB2 receptors, as well as 2 degrading enzymes, namely, fatty acid amide hydrolase and monoacylglycerol lipase, may correlate liver disease states with dysfunction of the endocannabinoid system. In all, the current stage of liver imaging using radioactive probes is still at its infant step; therefore, a new generation of target-specific imaging probes with a novel mechanism of action, proper uptake and washout kinetic, and reasonable metabolic profile, is still urgently needed in the molecular imaging field of liver diseases. We anticipate that there will be more efforts and advances in the development of novel imaging probes to focus on liver diseases, which ultimately would provide better diagnosis and prognosis in personalized medicine.

**Table 1.** Specific Molecular Tracers for NAFLD.

Molecular Probes	Molecular Targets	Liver Disease	SPECT/PET	Reference
[^18^F]FEDAC	TSPO	NASH	PET	^23^
		Liver fibrosis	PET	^24^
[^99m^Tc]cRGDfK	Integrin α_v_β_3_	Liver fibrosis	SPECT	^32^
[^99m^Tc]3PRGD_2_				^33^
[^18^F]FBHGal	ASGPRs	Liver fibrosis	SPECT	^39^
[^99m^Tc]MAMA-DGal				^40^
[^99m^Tc]p(VLA-co-VNI)				^41^
[^68^ Ga]NOTA-GSA	GSA	Liver fibrosis	PET	^44^
([^68^ Ga]DTPA-GSA				^45^
[^99m^Tc]GlcNAc-PEI	Desmin and vimentin	Liver fibrosis	SPECT	^48^

Abbreviations: ASGPR, asialoglycoprotein receptors; CT, computed tomography; GSA, galactosyl human serum albumin; NASH, nonalcoholic steatohepatitis; NAFLD, nonalcoholic fatty liver disease; PET, positron emission tomography; SPECT, single-photon emission computed tomography; TSPO, translocator protein 18 kDa.
